# Global longitudinal strain value for predicting left ventricular remodeling after primary percutaneous reperfusion therapy in acute myocardial infarction

**DOI:** 10.1186/cc14242

**Published:** 2015-03-16

**Authors:** JJ Jiménez, JL Iribarren, J Lacalzada, A De la Rosa, M Brouard, E Hurtado, S Diosdado, S Ramos, R Perez

**Affiliations:** 1 Hospital Universitario De Canarias, La Laguna, Spain

## Introduction

After an acute myocardial infarction with ST-segment elevation (STEMI) treated with percutaneous coronary intervention (PCI), the left ventricle (LV) can undergo negative remodeling (R-). We aimed to investigate whether global longitudinal strain (SGL) of the left ventricle (LV) predicts remodeling.

## Methods

Transthoracic echocardiography with speckle tracking imaging (TTE-STI) was performed 2 to 3 days after primary PCI and 6 months later in patients with diagnosis of STEMI. LV R- criteria were: LVEF increase ≤5% and end-diastolic volume increase ≥15%. Logistic regression and ROC curve analysis was used for the statistical analysis.

## Results

**Figure 1:**
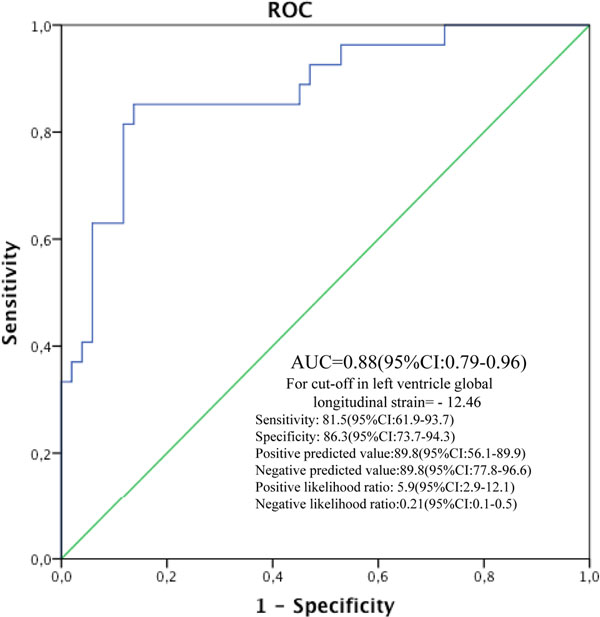


Eighty-three patients (56 ± 11 years) with STEMI at any LV localization and subjected to primary PCI were studied during 2012: LV R- patients (*n *= 35, 42%) and no LV R- patients (*n *= 48, 58%). Diabetes mellitus (41% vs. 19%; *P *< 0.001) and TnI levels (1.2 ± 2.1 μg/l vs. 0.4 ±0.3 μg/l; *P *= 0.005) showed higher incidence in LV R- patients. SGL was -12.5 ± 5.6% in no LV R- patients and -6.5 ± 3.4 in LV R- patients. In the regression analysis just LV SGL and SL in left anterior descending territory remained significant, OR: 1.85 (1.24 to 2.76) (*P *< 0.001) and OR: 1.63 (1.15 to 2.31) (*P *< 0.001), respectively. The analysis of ROC curves revealed that at the cutoff level of -12.46%, SGL identifies LV R- with a sensibility of 81% and a specificity of 86% (AUC = 0.88: 95% CI: 0.79 to 0.96; *P *< 0.001) (Figure 1).

## Conclusion

SGL assessment in the first days after primary PCI is useful in the prediction of LV R- independently of the myocardial infarction localization.

